# Association Between Rurality and Financial Performance of Public Hospitals in Japan: A Nationwide Cross‐Sectional Study

**DOI:** 10.1002/jgf2.70088

**Published:** 2025-12-08

**Authors:** Kota Sakaguchi, Takafumi Abe, Ayako Erabi, Tomotoshi Iseki, Kaneko Makoto, Yoshihiko Shiraishi, Takashi Watari

**Affiliations:** ^1^ General Medicine Center Shimane University Hospital Shimane Japan; ^2^ Center for Community‐Based Healthcare Research and Education (CoHRE) Head Office for Research and Academic Information, Shimane University Izumo Japan; ^3^ Faculty of Medicine Shimane University Shimane Japan; ^4^ Department of Management, Faculty of Business Administration Josai University Saitama Japan; ^5^ Department of Health Data Science Yokohama City University Yokohama Kanagawa Japan; ^6^ Sagamihara Endowed Chair in Comprehensive Community Medicine Kitasato University Sagamihara‐Shi Kanagawa Japan; ^7^ Integrated Clinical Education Center Kyoto University Hospital Kyoto Japan

**Keywords:** financial performance, health policy, healthcare disparities, public hospitals, rural health services, rurality index for Japan

## Abstract

**Background:**

Ensuring sustainable healthcare delivery in rural Japan is a policy priority. However, the relationship between geographic rurality, as measured objectively, and the financial performance of public hospitals essential to these areas remains underexplored.

**Objective:**

To examine the association between the Rurality Index for Japan (RIJ) and the likelihood of ordinary and medical service deficits in public hospitals, while adjusting for hospital size and bed occupancy rate.

**Methods:**

We conducted a nationwide cross‐sectional study using the fiscal year 2022 yearbook from Japan's Ministry of Internal Affairs and Communications. The primary outcomes were ordinary and medical service deficits, defined as a balance ratio of < 100%. Multivariable logistic regression was used to estimate adjusted odds ratios (aORs) and 95% confidence intervals (95% CIs) for RIJ quartiles, with hospital size and bed occupancy rate as covariates.

**Results:**

A total of 643 hospitals were analyzed. In unadjusted analyses, the highest rurality quartile (Q4) was associated with significantly higher odds of ordinary deficit (OR 1.79, 95% CI 1.11–2.88). However, in multivariable analyses, no statistically significant independent association was found between RIJ and either deficit. Conversely, larger hospitals (≥ 300 beds; aOR 0.53, 95% CI 0.32–0.89) and those with higher bed occupancy rates (≥ 65.6%) were significantly associated with lower odds of ordinary deficit.

**Conclusion:**

Hospital size and bed occupancy rate, rather than geographic rurality itself, are key structural factors associated with the financial sustainability of public hospitals in Japan.

## 1 Introduction

Japan's population has been declining since its peak in 2008, creating a demographic shift that has profoundly impacted the nation's socio‐economic fabric [1]. This trend is particularly pronounced in rural areas, such as mountainous regions and remote islands, where the dual challenges of depopulation and rapid population aging have created an urgent need for sustainable healthcare delivery systems [2, 3]. Private healthcare providers find it difficult to operate sustainably in these areas due to limited geographic accessibility to healthcare facilities, difficulties in recruiting and retaining healthcare professionals, and the inherent financial challenges posed by low population densities [4]. Consequently, public hospitals have long served as essential safety nets in rural Japan, delivering comprehensive services that include emergency care, perinatal care, pediatric care, mental health services, and disaster response [4]. However, despite their critical role in ensuring equitable access to healthcare, many of these public hospitals face chronic financial difficulties, as evidenced by persistent ordinary and medical service deficits [5].

The Japanese government has implemented the “Designated Non‐Profitable District Hospital” scheme to address the fiscal vulnerability of rural healthcare systems. This scheme provides financial support through local allocation of tax grants to hospitals that meet specific geographic and demographic criteria [6] to stabilize rural hospital operations. However, the binary administrative definitions used for eligibility may not adequately capture the nuanced and multidimensional gradients of rurality and the corresponding operational challenges faced by hospitals in these areas. The lack of a standardized, objective measure of rurality is a major limitation in healthcare policy and planning in Japan [2].

To overcome the limitations of administrative definitions, the rurality index for Japan (RIJ)—a composite and continuous metric—was developed to provide a standardized, objective measure of rurality [7]. The RIJ has proven useful in various healthcare studies [8, 9, 10, 11]. However, despite its promising utility, no studies have comprehensively examined the association between the RIJ and the financial performance of public hospitals. Given the critical role of these hospitals in sustaining rural healthcare systems, clarifying how objectively measured rurality relates to their financial sustainability is essential for designing effective and equitable policy interventions.

## 2 Objective

To examine the association between rurality, as measured by the RIJ, and the likelihood of financial deficits (specifically, ordinary and medical service deficits) in public hospitals across Japan.

## 3 Methods

### 3.1 Study Design

This nationwide cross‐sectional observational study was designed to evaluate the association between rurality and financial indicators among public hospitals in Japan during the 2022 fiscal year. The manuscript adheres to the Strengthening the Reporting of Observational Studies in Epidemiology guidelines for reporting observational studies [12].

### 3.2 Study Setting

Japan's healthcare system is characterized by a statutory health insurance system that provides universal coverage and allows patients free access to any medical institution. The system comprises a mix of public and private healthcare providers, with the private sector making up the majority of facilities. The reimbursement system is based on a nationally uniform fee‐for‐service schedule, although a prospective payment system (the diagnosis procedure combination/per‐diem payment system) is widely used for acute inpatient care [13, 14]. Within this context, public hospitals, operated by local governments, are mandated to function as a crucial safety net. They are legally tasked with providing essential but potentially unprofitable services—such as emergency, perinatal, pediatric, and disaster medicine—particularly in medically underserved areas where private sector participation may be limited [4, 14].

### 3.3 Data Source and Study Population

The primary data source was the 2022 Fiscal Year (FY) Yearbook of Local Public Enterprises (Chiho Koei Kigyo Nenkan) published by the Ministry of Internal Affairs and Communications, Japan [15]. This dataset contains detailed information on the financial performance, operational status, and facility characteristics of all public hospitals operated by local governments (prefectural and municipal) in Japan for FY2022.

The study population included all public hospitals listed in the FY2022 Yearbook. We conducted a complete case analysis, including only hospitals with the following data: (1) data necessary to determine the primary outcomes—ordinary and medical service deficits (derived from the ordinary and medical balance ratios); (2) the main explanatory variable—RIJ, calculated based on the hospital's postal code; and (3) the covariates—total number of hospital beds and general bed occupancy rate. Hospitals with missing information or unclear location data that precluded variable calculation were excluded from the analysis.

### 3.4 Variables and Definitions

*Primary Outcomes*: The primary outcomes were two key financial indicators: ordinary and medical service deficits. These were defined based on the ordinary and medical balance ratios reported in the FY2022 Yearbook. An ordinary balance ratio of < 100% was categorized as an ordinary deficit, and a medical balance ratio of < 100% was categorized as a medical service deficit. Ratios of ≥ 100% were considered balanced or surplus [16, 17].

### 3.5 Main Explanatory Variable

Rurality was assessed using the RIJ [7]. The RIJ is a composite measure based on postal code units, incorporating four geographic and demographic factors: straight‐line distance to the nearest advanced emergency medical center, population density, island status, and designation as a special heavy snowfall area. The RIJ score ranges from 1 (most urban) to 100 (most rural). In descriptive analyses, the RIJ was treated as a continuous variable. For logistic regression models, the RIJ was categorized into quartiles (Q1: least rural to Q4: most rural).

### 3.6 Covariates

Covariates included two key factors previously reported to be associated with hospital financial performance: (1) Total number of hospital beds, categorized into five groups: < 100, 100–149, 150–199, 200–299, and ≥ 300 beds [6, 17], and (2) General bed occupancy rate, categorized into quartiles based on the distribution of the study sample: Q1 (≤ 54.2%), Q2 (54.3%–65.5%), Q3 (65.6%–74.4%), and Q4 (≥ 74.5%). These variables were selected based on previous studies on hospital financial performance and reports from the Ministry of Internal Affairs and Communications [6, 7, 16, 17].

### 3.7 Statistical Analysis

All statistical analyses were conducted using Stata/SE 17.0 (StataCorp, College Station, TX, USA). Descriptive statistics are presented as means and standard deviations or medians and interquartile ranges for continuous variables and as frequencies and percentages for categorical variables. For the characteristics (Table 1), differences across RIJ quartiles were assessed using the chi‐squared test for categorical variables and analysis of variance for continuous variables. Univariable logistic regression was used to examine the crude association between each variable (RIJ quartiles, bed count categories, and occupancy rate categories) and the outcomes (ordinary and medical service deficits). Multivariable logistic regression models were constructed by including all variables. The results are reported as odds ratios (ORs), adjusted odds ratios (aORs), 95% confidence intervals (CIs), and *p*‐values. As a complementary analysis, a multivariable linear regression was conducted treating the medical service balance ratio as a continuous outcome. To address skewness, the dependent variable was log‐transformed prior to modeling. The regression model included RIJ quartiles, number of beds, and bed occupancy rate quartiles as covariates. A two‐sided *p*‐value of < 0.05 was considered statistically significant.

**TABLE 1:** Characteristics of the included public hospitals, stratified by Rurality Index for Japan quartiles.

Characteristic	Overall (*N* = 643)	Q1: Most urban (*N* = 161)	Q2: Moderately urban (*N* = 183)	Q3: Moderately rural (*N* = 147)	Q4: Most rural (*N* = 152)	*p*
RIJ score, mean ± SD	44.9 ± 23.9	20.1 ± 8.5	36.1 ± 3.0	44.1 ± 2.9	82.7 ± 12.3	
Number of beds, *n* (%)						
< 100	212 (33.0)	12 (7.5)	34 (18.6)	63 (42.9)	103 (67.8)	< 0.001
100–149	81 (12.6)	12 (7.5)	27 (14.8)	16 (10.9)	26 (17.1)	
150–199	81 (12.6)	21 (13.0)	25 (13.7)	24 (16.3)	11 (7.2)	
200–299	65 (10.1)	17 (10.6)	25 (13.7)	17 (11.6)	6 (3.9)	
≥ 300	204 (31.7)	99 (61.5)	72 (39.3)	27 (18.4)	6 (3.9)	
Bed occupancy rate, *n* (%)						
Q1: ≤ 54.2%	161 (25.0)	20 (12.4)	40 (21.9)	38 (25.9)	63 (41.4)	< 0.001
Q2: 54.3%–65.5%	161 (25.0)	51 (31.7)	50 (27.3)	31 (21.1)	29 (19.1)	
Q3: 65.6%–74.4%	161 (25.0)	42 (26.1)	56 (30.6)	39 (26.5)	24 (15.8)	
Q4: ≥ 74.5%	160 (24.9)	48 (29.8)	37 (20.2)	39 (26.5)	36 (23.7)	
Ordinary balance, *n* (%)						
Surplus	421 (65.5)	118 (73.3)	121 (66.1)	90 (61.2)	92 (60.5)	0.066
Deficit	222 (34.5)	43 (26.7)	62 (33.9)	57 (38.8)	60 (39.5)	
Medical service balance, *n* (%)						
Surplus	18 (2.8)	9 (5.6)	4 (2.2)	3 (2.0)	2 (1.3)	0.094
Deficit	625 (97.2)	152 (94.4)	179 (97.8)	144 (98.0)	150 (98.7)	

*Note:* *p*‐values were calculated using chi‐squared test for categorical variables and ANOVA for continuous variables. Data are presented as mean ± SD or *n* (%). RIJ quartiles: Q1 (most urban, RIJ score 1–30), Q2 (moderately urban, RIJ score 31–40), Q3 (moderately rural, RIJ score 41–50), Q4 (most rural, RIJ score 51–100). Bed occupancy rate quartiles: Q1 (≤ 54.2%), Q2 (54.3%–65.5%), Q3 (65.6%–74.4%), Q4 (≥ 74.5%).

Abbreviations: ANOVA, analysis of variance; RIJ, Rurality Index for Japan; SD, standard deviation.

### 3.8 Ethical Considerations

This Institutional Review Board of Shimane University Hospital approved this study (Approval Number: KS20240517‐2). This study used publicly available, anonymized data. Hence, the requirement for informed consent was waived.

## 4 Results

### 4.1 Study Population

**FIGURE 1:**
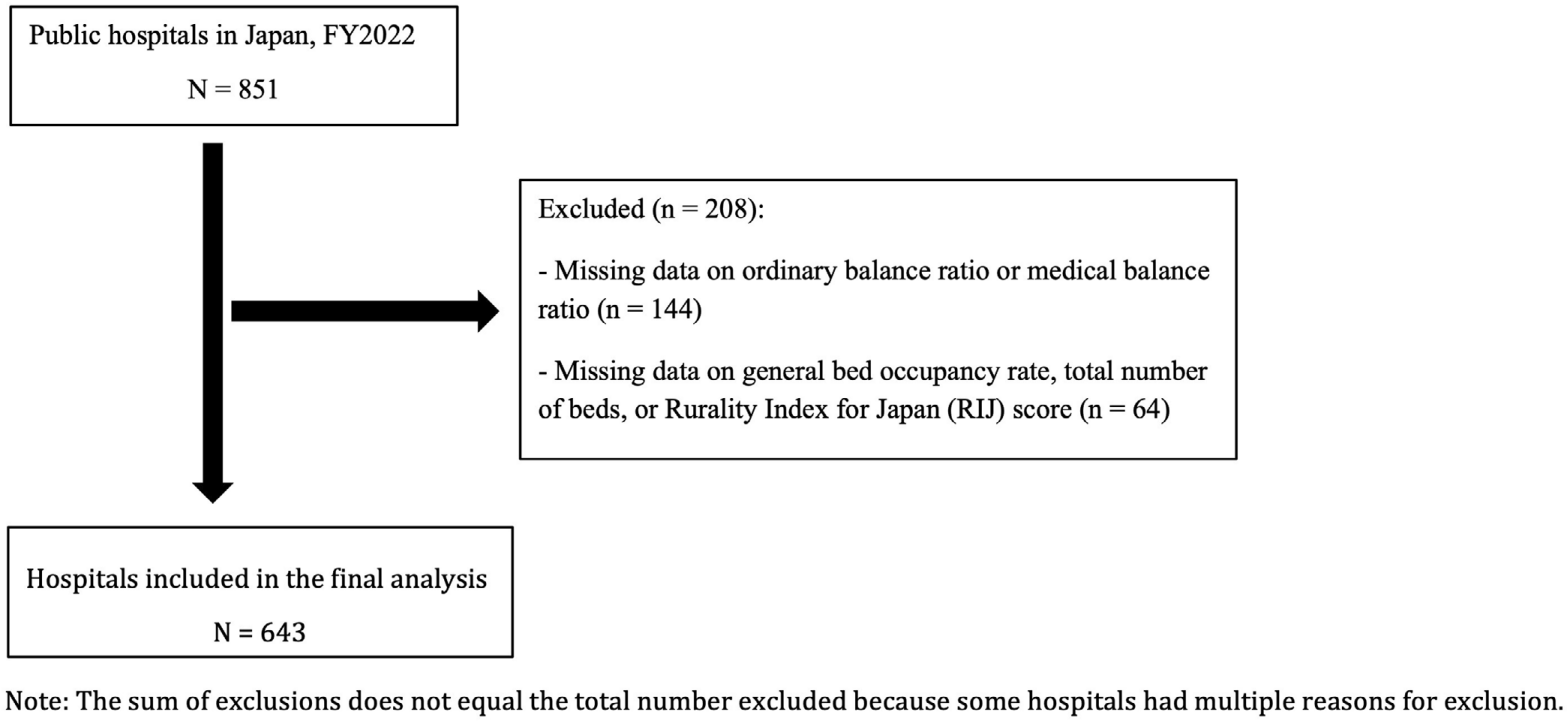
Flowchart of hospital selection.

The initial study population comprised 851 public hospitals operating in Japan during FY2022. Of these, we excluded 44 hospitals due to missing data on the ordinary balance ratio and 144 due to missing data on the medical balance ratio (with some overlap). In addition, hospitals with missing data on key variables, including the RIJ, total bed count, or general bed occupancy rate, and those with unclear location information were excluded. After applying these criteria, we included 643 hospitals in the final analysis (Figure 1).

### 4.2 Characteristics of Included Hospitals

Table 1 presents the characteristics of the 643 included hospitals stratified by RIJ quartiles (Q1: most urban to Q4: most rural). The mean (± standard deviation) RIJ score was 20.1 (±8.5) and 82.7 (±12.3) in Q1 and Q4, respectively, demonstrating a clear gradient across quartiles. Regarding hospital size, 61.5% of Q1 hospitals had ≥ 300 beds, whereas 67.8% of Q4 hospitals were small‐scale facilities with < 100 beds, indicating a strong negative correlation between rurality and hospital size. For bed occupancy rate, 41.4% of Q4 hospitals fell into the lowest occupancy quartile (≤ 54.2%), indicating a tendency for lower occupancy rates in more rural areas. The proportion of hospitals with an ordinary deficit increased with rurality, from 26.7% in Q1 to 39.5% in Q4. In contrast, the prevalence of medical service deficits was high across all rurality levels, affecting 97.2% of all hospitals (Table 1).

### 4.3 Associations Between Rurality and Financial Deficits: Logistic Regression Analysis

Logistic regression analyses were conducted to examine the associations between RIJ quartiles and ordinary and medical service deficits. The multivariable models were adjusted for total bed count and general bed occupancy rate (Table 2).

**TABLE 2:** Factors associated with ordinary and medical service deficits: Univariable and multivariable logistic regression analyses.^a^

	Ordinary deficit						Medical service deficit					
Variable	Univariable analysis			Multivariable analysis			Univariable analysis			Multivariable analysis		
	OR	95% CI	*p*	aOR	95% CI	*p*	OR	95% CI	*p*	aOR	95% CI	*p*
RIJ	1	Reference		1	Reference		1	Reference		1	Reference	
Q1: Most urban												
Q2: Moderately urban	1.41	0.88–2.24	0.15	1.22	0.75–1.98	0.421	2.65	0.80–8.77	0.111	2.8	0.81–9.62	0.102
Q3: Moderately rural	1.74	1.07–2.81	0.024	1.31	0.77–2.22	0.323	2.84	0.75–10.71	0.123	3.1	0.75–12.74	0.117
Q4: Most rural	1.79	1.11–2.88	0.017	1.12	0.63–1.98	0.698	4.44	0.94–20.90	0.059	4.14	0.74–23.14	0.106
Number of beds	1	Reference		1	Reference		1	Reference		1	Reference	
< 100												
100–149	0.62	0.36–1.06	0.079	0.68	0.39–1.18	0.168	0.37	0.07–1.89	0.233	0.55	0.10–2.99	0.492
150–199	0.81	0.48–1.37	0.427	0.85	0.49–1.49	0.575	0.28	0.06–1.26	0.097	0.42	0.08–2.13	0.298
200–299	0.76	0.43–1.35	0.355	0.85	0.46–1.58	0.601	0.45	0.07–2.77	0.39	0.74	0.11–5.03	0.762
≥ 300	0.41	0.27–0.63	< 0.001	0.53	0.32–0.89	0.016	0.47	0.12–1.92	0.295	1.34	0.27–6.60	0.722
Bed occupancy rate quartile	1	Reference		1	Reference		1	Reference		1	Reference	
Q1: ≤ 54.2%												
Q2: 54.3%–65.5%	0.66	0.42–1.04	0.071	0.78	0.49–1.24	0.287	0.25	0.03–2.22	0.211	0.32	0.03–3.00	0.319
Q3: 65.6%–74.4%	0.42	0.26–0.67	< 0.001	0.52	0.32–0.85	0.009	0.16	0.02–1.36	0.093	0.17	0.02–1.51	0.112
Q4: ≥ 74.5%	0.45	0.28–0.71	0.001	0.54	0.33–0.87	0.011	0.14	0.02–1.12	0.064	0.15	0.02–1.27	0.082

*Note:* Reference groups: RIJ Q1 (Most urban, RIJ score 1–30) for RIJ quartiles; < 100 beds for Number of beds; Q1 (≤ 54.2%) for Bed occupancy rate quartile.

Abbreviations: aOR, adjusted odds ratio; CI, confidence interval; OR, odds ratio; RIJ, Rurality Index for Japan.

^a^ Multivariable models were adjusted for RIJ quartile, number of beds category, and bed occupancy rate quartile.

### 4.4 Ordinary Deficit

In the univariable analysis, hospitals located in moderately rural areas (Q3) and highly rural areas (Q4) had significantly higher odds of an ordinary deficit than urban hospitals (Q1) (Q3: OR 1.74, 95% CI 1.07–2.81, *p* = 0.024; Q4: OR 1.79, 95% CI 1.11–2.88, *p* = 0.017). However, after adjusting for covariates, we observed no significant association between the RIJ quartile and ordinary deficit. In contrast, hospital size and bed occupancy rate showed significant associations. Hospitals with ≥ 300 beds had significantly lower adjusted odds of an ordinary deficit than those with < 100 beds (aOR 0.53, 95% CI 0.32–0.89, *p* = 0.016). Similarly, hospitals with higher occupancy rates demonstrated lower adjusted odds of an ordinary deficit: Q3 (65.6%–74.4%, aOR 0.52, 95% CI 0.32–0.85, *p* = 0.009) and Q4 (≥ 74.5%, aOR 0.54, 95% CI 0.33–0.87, *p* = 0.011), than the lowest occupancy quartile (Q1: ≤ 54.2%).

### 4.5 Medical Service Deficit

In the univariable analysis, hospitals in the most rural areas (Q4) had higher odds of a medical service deficit than urban hospitals (Q1) (OR 4.44, 95% CI 0.94–20.90, *p* = 0.059), although this was not statistically significant. A trend toward lower odds of a medical service deficit was observed for hospitals with 150–199 beds compared to those with < 100 beds (OR 0.28, 95% CI 0.06–1.26, *p* = 0.097). Hospitals in the highest occupancy quartile (Q4: ≥ 74.5%) also showed a non‐significant trend toward lower odds of a medical service deficit compared to the lowest quartile (Q1) (OR 0.14, 95% CI 0.02–1.12, *p* = 0.064). These trends persisted in multivariable analyses, but no statistically significant associations were found between any variables and the occurrence of a medical service deficit.

As a complementary analysis to address the high prevalence of deficits, we performed a multivariable linear regression analysis using the medical service balance ratio as a continuous variable. The results, presented in Table S1, showed no statistically significant association between RIJ quartiles and hospital size (*p* > 0.05). Conversely, hospitals with higher bed occupancy rates showed a strong and significant association with better financial outcomes (Q2: *p* = 0.001, Q3: *p* < 0.001, Q4: *p* = 0.004). No statistically significant association was found between hospital size and the continuous medical service balance ratio.

## 5 Discussion

This cross‐sectional study examined the association between the RIJ and key financial performance indicators among public hospitals in Japan, while adjusting for potential confounders such as hospital size and bed occupancy rate. The main findings revealed that higher rurality scores were associated with a higher likelihood of financial deficits in unadjusted analyses, although these associations did not remain statistically significant after controlling for structural factors, such as hospital size and occupancy. Conversely, larger hospital size (≥ 300 beds) and higher bed occupancy rates (≥ 65.6%) were significantly associated with a lower likelihood of ordinary deficit in adjusted analyses.

### 5.1 Interpretation of Findings in Context

A key finding of this study is that the initial association between higher rurality and a higher prevalence of ordinary deficit was attenuated after adjusting for structural factors. This suggests that the apparent association may be explained by the fact that hospitals in more rural areas tend to be smaller and have lower occupancy rates. Thus, geographic rurality may serve as a proxy for these underlying structural vulnerabilities rather than being an independent determinant of financial outcomes [18, 19]. However, an alternative interpretation must be considered. These structural factors might not be classical confounders but rather mediators on the causal pathway from rurality to financial deficits. From this perspective, rurality is associated with smaller hospital size and lower occupancy, which in turn contributes to financial strain. If this is the case, our adjustment for these variables may have underestimated the true total effect of rurality on financial performance.

### 5.2 Financial Indicators

Nearly all hospitals in this study (97.2%) experienced medical service deficits, with no statistically significant associations detected between rurality and medical service deficits in the multivariate model. However, the direction of the odds ratios suggested a trend toward a higher occurrence in higher‐rurality areas, albeit with wide confidence intervals, indicating potential residual confounding by factors such as small catchment populations and difficulty in aggregating case volume [20, 21]. The lack of significant findings for medical service deficits may reflect limitations in the current model, which did not include key variables such as specialty mix, personnel costs, and supply costs—factors known to be associated with medical profitability [22].

The differential associations between rurality and the two financial indicators—ordinary and medical service deficits—may stem from the distinct nature of these measures. Ordinary deficits are influenced by government subsidies and fiscal transfers, which can partially offset the challenges faced in rural areas. In contrast, medical service deficits more directly reflect clinical revenue streams and operational efficiency [6]. These findings suggest that while structural subsidies may mitigate rural disadvantages in operating budgets, further analyses are warranted to explore mechanisms related to medical service deficits in rural hospitals. The lack of statistically significant findings for medical service deficits may also be attributed to the extremely high prevalence of deficits (97.2%), which can limit the statistical power of logistic regression analysis. While we focused on the binary outcome of deficit versus surplus to align with common policy indicators, we acknowledge the statistical challenge this poses. As detailed in the Results section, we conducted a preliminary linear regression analysis using the medical balance ratio as a continuous variable, which also showed similar trends but no statistically significant associations with RIJ after adjusting for structural factors. This suggests that future research might explore more sophisticated statistical models to better capture the nuances of medical profitability in this context.

### 5.3 Hospital Size and Bed Occupancy

In this study, a significant association was observed between ordinary deficit and bed occupancy. In addition, regarding the relationship between medical service deficits and bed occupancy, no significant association was observed in the logistic regression analysis. However, when the outcome was treated as a continuous variable in a multiple linear regression model, a positive association was identified. Although medical service deficits could not be suppressed, the findings suggest that improving medical balance ratios may be a feasible approach. The inverse associations observed between larger hospital size and higher bed occupancy and the likelihood of ordinary deficit, underscore the critical role of scale and efficiency in public hospital management [23]. Similarly, previous studies have demonstrated that improving bed occupancy rates contributes to better financial outcomes [24], and hospitals above certain size thresholds benefit from economies of scale [25]. These findings highlight the importance of targeted strategies to improve occupancy, including optimizing staff allocation, enhancing regional collaborations, and leveraging telemedicine to improve access [26, 27]. Notably, studies from the United States have also reported severe financial challenges in small rural hospitals [28], suggesting that the relationship between geographic barriers and hospital sustainability is a global concern [29]. Japan's current designation of “non‐profitable area hospitals” based on administrative units has been criticized for not accurately reflecting the true gradient of the geographic disadvantage [30]. A more robust approach for policy decisions would be to use an index like the RIJ, whose validity is supported by its demonstrated association with clinical outcomes, such as in acute stroke care. Using a clinically validated index would allow for a more nuanced and equitable approach to policy reforms. This present study extends prior work on regional healthcare resource distribution by providing empirical evidence on financial conditions in rural hospitals [31].

## 6 Policy Implications and Future Directions

These findings have important policy implications. First, to move beyond blunt administrative designations, policymakers should incorporate continuous and objective indices, such as the RIJ to enable more precise and equitable targeting of financial support. Second, our results underscore the urgent need for strategies to improve bed occupancy, such as strengthening regional collaborations. Third, the financial vulnerability of rural hospitals cannot be addressed solely through internal management; comprehensive policy support, including rural‐specific reimbursement enhancements and expanded grant programs, is essential [32, 33].

## 7 Limitations

This study has some limitations. First, its cross‐sectional design precludes causal inference; for instance, whether low occupancy leads to financial deficits or if financially distressed hospitals struggle to maintain occupancy remains unclear. Second, the analyses relied on a single‐year dataset (FY2022), and the findings may not be fully generalizable to a typical fiscal year due to the unique financial support and patient volume fluctuations of the COVID‐19 pandemic. According to reports from the Ministry of Internal Affairs and Communications [15, 16], special subsidies were provided to stabilize hospital management during this period, which may have influenced the balance ratios. It is plausible that the impact of these subsidies and the concurrent patient volume changes may have differed between rural and urban areas, potentially masking the true underlying financial vulnerabilities of rural hospitals. Further research with multi‐year data is warranted to fully disentangle the effects of the pandemic from the long‐term trends in financial performance. Third, while the RIJ captures geographic factors, it does not account for variables such as population demographics, workforce composition, or management quality; omitting these factors could lead to residual confounding. Fourth, excluding hospitals with missing data may have introduced selection bias. If data were missing completely at random, the impact would be minimal. However, if smaller, more financially strained hospitals were systematically less likely to provide complete data, our results might underestimate the prevalence of financial deficits in rural areas. Fifth, in our analytical model, hospital size and bed occupancy rate were treated as confounders. An alternative perspective, however, is that these variables could represent a component of the pathway through which rurality is associated with financial outcomes. If so, adjusting for these factors might obscure the overall association between rurality and financial performance. Therefore, further investigation using different analytical methods is warranted to better understand the complex interplay between rurality, hospital structure, and financial status. Finally, the inclusion of subsidies in the medical balance ratio data may have obscured the “actual” operational profitability of hospitals, making it harder to identify factors associated purely with clinical revenue and expenses.

## 8 Conclusion

This nationwide cross‐sectional study examined the association between the RIJ and financial deficits (ordinary and medical service deficits) in public hospitals, while adjusting for hospital size and bed occupancy rate. Higher rurality scores were associated with a higher likelihood of deficits in unadjusted analyses; however, these associations were not statistically significant after controlling for structural factors. In contrast, larger hospital size (≥ 300 beds) and higher bed occupancy (≥ 65.6%) were significantly associated with a lower likelihood of ordinary deficit. These findings suggest that structural and modifiable factors such as hospital size and efficiency play a greater role than geographic rurality in determining the financial status of public hospitals. Sustainable rural healthcare systems will require targeted interventions to improve operational efficiency and flexible policy frameworks that reflect the complex realities of rural healthcare delivery.

## Author Contributions

Kota Sakaguchi: conceptualization, methodology, data curation, formal analysis, writing – original draft, project administration, supervision. Takafumi Abe: methodology, writing – review and editing, supervision. Ayako Erabi: data curation, investigation, writing – review and editing. Tomotoshi Iseki: writing – review and editing, supervision. Makoto Kaneko: methodology, writing – review and editing, supervision. Yoshihiko Shiraishi: writing – review and editing, supervision. Takashi Watari: supervision, writing – review and editing.

## Funding

This research was supported in part by the Japan Society for the Promotion of Science (JSPS) KAKENHI Grant‐in‐Aid for Scientific Research [Grant Number: 24K13364].

## Ethics Statement

This Institutional Review Board of Shimane University Hospital approved this study (Approval Number: KS20240517‐2). This study used publicly available, anonymized data. Hence, the requirement for informed consent was waived.

## Consent

Patient consent was not required for this study because it involved a retrospective analysis of publicly available, anonymized data, and this was waived by the Institutional Review Board of Shimane University Hospital.

## Conflicts of Interest

Dr. Watari, Takashi is an Editorial Board member of JGFM Journal and a co‐author of this article. To minimize bias, they were excluded from all editorial decision‐making related to the acceptance of this article for publication.

## Supporting information


Table S1.

## Data Availability

Data supporting this study's findings are publicly available from the “Fiscal Year 2022 Yearbook of Local Public Enterprises (Chiho Koei Kigyo Nenkan)” published by the Ministry of Internal Affairs and Communications, Japan. These data can be accessed at https://www.soumu.go.jp/main_sosiki/c‐zaisei/hospital/kessan‐bunseki/R04.html. Further derived data supporting the findings of this study are available from the corresponding author upon reasonable request.
