# Supplementary material for: Association Between Rurality and Financial Performance of Public Hospitals in Japan: A Nationwide Cross‐Sectional Study
Source: J Gen Fam Med. 2025 Dec 8;27(1):e70088. doi: 10.1002/jgf2.70088 (PMC12897556; doi:10.1002/jgf2.70088)
Supplement: Supplementary file 1 — Table S1. [file JGF2-27-e70088-s001.docx]

Supplemental file

Title: Supplemental Table S1. Results of Linear Regression Analysis for Medical Service Balance Ratio

| Variable | Coefficient | Std err. | t-value | p-value | 95% CI |
| --- | --- | --- | --- | --- | --- |
| RIJ | Reference |  |  |  |  |
| Q1:Most urban |  |  |  |  |  |
| Q2:Moderately urban | -0.001 | 0.081 | -0.01 | 0.993 | -0.160–0.158 |
| Q3:Moderately rural | 0.053 | 0.090 | 0.59 | 0.558 | -0.160–0.231 |
| Q4:Most rural | -0.009 | 0.098 | -0.09 | 0.930 | -0.202–0.185 |
| Number of beds | Reference |  |  |  |  |
| <100 |  |  |  |  |  |
| 100–149 | 0.034 | 0.098 | 0.35 | 0.725 | -0.158–0.227 |
| 150–199 | -0.133 | 0.101 | -1.32 | 0.186 | -0.331–0.064 |
| 200–299 | 0.006 | 0.110 | 0.06 | 0.954 | -0.211–0.224 |
| ≥300 | 0.100 | 0.089 | 1.12 | 0.264 | -0.075–0.275 |
| Bed occupancy rate quartile | Reference |  |  |  |  |
| Q1:≤54.2% |  |  |  |  |  |
| Q2:54.3–65.5% | 0.274 | 0.085 | 3.23 | **0.001** | 0.108–0.440 |
| Q3:65.6–74.4% | 0.341 | 0.086 | 3.95 | **0.000** | 0.171–0.510 |
| Q4: ≥74.5% | 0.245 | 0.084 | 2.90 | **0.004** | 0.079–0.410 |

Notes. Multivariable linear regression analysis was performed using the log-transformed medical service balance ratio as a continuous variable. The model included RIJ quartiles, number of beds, and bed occupancy rate quartiles as covariates. This analysis was conducted on the same sample of 643 hospitals as the primary analysis. The reference groups for the analysis are Q1 RIJ, <100 beds, and Q1 bed occupancy rate. The analysis shows no statistically significant association between RIJ and the log-transformed balance ratio. However, significant associations are found for the "Q2, Q3, and Q4" bed occupancy rate categories, suggesting that hospital efficiency is a key factor in medical financial performance.

Legends. RIJ, Rurality Index for Japan; CI, confidence interval. Q1-Q4, Quartiles, with Q1 being the most urban and Q4 being the most rural. Beds, Total number of hospital beds. Bed occupancy rate, General bed occupancy rate.
